# Alpha band disruption in the AD-continuum starts in the Subjective Cognitive Decline stage: a MEG study

**DOI:** 10.1038/srep37685

**Published:** 2016-11-24

**Authors:** D. López-Sanz, R. Bruña, P. Garcés, C. Camara, N. Serrano, I. C. Rodríguez-Rojo, M. L. Delgado, M. Montenegro, R. López-Higes, M. Yus, F. Maestú

**Affiliations:** 1 Laboratory of Cognitive and Computational Neuroscience, Center for Biomedical Technology, Complutense University of Madrid and Technical University of Madrid, Spain; 2 Department of Basic Psychology II, Complutense University of Madrid, Spain; 3 Memory Decline Prevention Center Madrid Salud, Ayuntamiento de Madrid, Spain; 4 Radiology Department, San Carlos University Hospital, Madrid, Spain

## Abstract

The consideration of Subjective Cognitive Decline (SCD) as a preclinical stage of AD remains still a matter of debate. Alpha band alterations represent one of the most significant changes in the electrophysiological profile of AD. In particular, AD patients exhibit reduced alpha relative power and frequency. We used alpha band activity measured with MEG to study whether SCD and MCI elders present these electrophysiological changes characteristic of AD, and to determine the evolution of the observed alterations across AD spectrum. The total sample consisted of 131 participants: 39 elders without SCD, 41 elders with SCD and 51 MCI patients. All of them underwent MEG and MRI scans and neuropsychological assessment. SCD and MCI patients exhibited a similar reduction in alpha band activity compared with the no SCD group. However, only MCI patients showed a slowing in their alpha peak frequency compared with both SCD and no SCD. These changes in alpha band were related to worse cognition. Our results suggest that AD-related alterations may start in the SCD stage, with a reduction in alpha relative power. It is later, in the MCI stage, where the slowing of the spectral profile takes place, giving rise to objective deficits in cognitive functioning.

Alzheimer’s Disease (AD) is a growing threat nowadays. Every year around 4.6 million new AD cases are diagnosed all over the world and the number of people that will be affected by 2040 is estimated around 81 million^1^. The vast popularity of the amyloid cascade hypothesis has led therapeutic efforts to focus on removing or inhibiting the Aβ plaques formation. However, none of them have shown any improvement so far. This led to a shift in research focus towards the initial stages of AD. The progression of the disease is slow and insidious and the preclinical stage may last several years and start even decades before the first dementia symptoms show up^2^. Targeting individuals in the asymptomatic at-risk stage of AD with prevention trials may be the most effective approach^3^ hence there is a growing interest in detecting as early as possible the first signs of AD pathology.

This preclinical stage has been alluded to with different terms in the literature, but it was recently named as Subjective Cognitive Decline (SCD) and a common conceptual framework was settled for research by the SCD-Initiative^4^. This stage is characterized by a normal performance on neuropsychological tests besides the subjective perception of worsening in the cognitive functioning. It is also remarkable the presence of AD biomarkers with a very subtle cognitive decline, which can be still compensated thus remaining undetectable by standard neuropsychological testing. However, the idea of SCD representing a very early preclinical stage of the AD continuum has been controversial as memory concerns are highly associated with psychological factors such as personality traits, anxiety or depression^5^,^6^.

However, a growing body of literature seems to support that SCD could be an indicator of early AD pathology. The presence of self-perceived decline has been associated with subsequent cognitive decline and progression to dementia^7^. Additionally, people with SCD present greater rates of incident mild cognitive impairment and dementia compared to normal elders without any perceived cognitive concern^8^,^9^, and its conversion rate per year to mild cognitive impairment (MCI) and dementia is around 6.6% and 2.3% respectively^10^. Cerebrospinal fluid (CSF) analysis also supports this AD-spectrum hypothesis: SCD subjects present AD-related markers in the CSF (i.e. decreased Aβ_42_ and increased tau)^11^. Nevertheless, there are still some inconsistent results: while some groups reported reduced hippocampal volume in patients with SCD^12^,^13^, others failed to find differences between SCD and age-matched controls^14^. Similar discrepancies have been reported with regard to Aβ depositions in the brain; although some studies showed higher levels of Aβ accumulation^12^,^15^ there are some others reporting negative results^16^.

Very little is known about the functional organization of the brain in this stage. Participants with SCD exhibited greater brain BOLD responses in fMRI compared to controls in several different paradigms; during memory^17^, and attentional tasks^18^, and also increased resting state connectivity in the Default Mode Network (DMN)^19^.

EEG and MEG have been proven useful in characterizing different stages of the AD continuum^20^,^21^. The most characteristic finding regarding the spectral profile of AD is the slowing of the brain rhythms^22^. A slowing of the alpha peak has been reported in AD^23^ and MCI^24^. Furthermore, mean alpha frequency in MEG power spectra is reduced in MCI patients indicating that the beginning of this slowing may take place in the pre-dementia stage^25^. MCI patients, both amnestic single and multi-domain show a similar pattern, with an increase in delta and theta power and decremented alpha and beta power^21^,^26^,^27^. Alpha rhythm is of crucial importance. It is the predominant rhythm in the resting brain dynamics and it can be measured over wide regions in the human brain. Besides the power of the band, its main frequency can be easily identified given its prominent nature in the human power spectrum. These two values can draw together an overview of the power spectrum of each group, as alpha peak is the most prominent peak in human power spectrum, thus giving an insight about the time-course of the ‘shift to the left’ effect described in the AD electrophysiological profile.

Considering all these findings, the use of MEG to study neural dynamics in SCD could provide some insight into the matter. If SCD is actually one of the first stages of dementia, alpha activity could present alterations in line of those reported for MCI and AD (i.e. alpha power decrease or/and alpha slowing). As above mentioned, those neuroimaging studies reporting brain alterations in SCD have mostly reported AD-like pathology or changes in a similar direction but to a lesser extent to those exhibited by AD patients. However, to the best of our knowledge the only study to date reporting resting state power spectral analysis in SCD reported an increase in alpha power both in eyes open and eyes closed condition with respect to controls without cognitive concerns^28^. The authors suggested a nonlinear evolution from healthy controls to AD. However, this is not consistent with different studies reporting AD-like changes in SCD: progressive reduced brain metabolism^15^, progressive increased amyloid burden^12^ or brain atrophy^29^.

The present study aims to elucidate two main points; (1) whether SCD stage can be considered a pre-dementia stage in which alpha band patterns are disrupted and (2) given the first, we seek to determine the direction of the observed changes (i.e. whether changes follow a linear continuous with MCI-AD or a non-linear pattern). For this purpose we use MEG data from healthy elders without any cognitive concern (no SCD), healthy elders with Subjective Cognitive Decline (SCD) and elders with Mild Cognitive Impairment (MCI). Alpha power and peak parameters were computed in source space for a better spatial characterization of the alpha rhythm pattern in every group. With the specified sample groups we can assess and describe the changes and the evolution of the alpha power spectrum occurring along all the pre-dementia stages characterized to date.

## Materials and Methods

### Subjects

The sample of this study was recruited from three different sources; the Neurology Department in “Hospital Universitario San Carlos”, the “Center for Prevention of Cognitive Impairment” and the “Seniors Center of Chamartin District” located in Madrid (Spain). All participants were right-handed and native Spanish speakers.

The sample consisted of 131 elders. 51 of them were catalogued as mild cognitive impairment (MCI group), while 80 showed no objective neuropsychological impairment. The latter were further divided in two groups, 39 elders without any cognitive concern (no SCD group) and 41 with subjective cognitive decline (SCD group). Table 1 summarizes their demographic data and other relevant characteristics.

### Diagnostic criteria

In order to assess the general cognitive and functioning status in the sample a set of screening questionnaires were administered to every participant: The Mini Mental State Examination (MMSE^30^), the Geriatric Depression Scale – Short Form (GDS-SF^31^), the Hachinski Ischemic Score (HIS^32^) and the Functional Assessment Questionnaire (FAQ^33^).

After the initial screening all subjects underwent an exhaustive neuropsychological assessment including: Direct and Inverse Digit Span Test (Wechsler Memory Scale, WMS-III),Immediate and Delayed Recall (WMS-III), Phonemic and Semantic Fluency (Controlled oral Word Association Test, COWAT), Ideomotor Praxis of Barcelona Test, Boston Naming Test (BNT) and Trail Making Test A and B (TMTA and TMTB)^34^ and Rule Shift Cards (Behavioural Assessment of the Dysexecutive Syndrome, BADS).

Subjects were diagnosed as MCI according to the criteria established by Petersen^35^ and Grundman^36^. MCI patients did not fulfill criteria for dementia diagnosis.

Cognitive concerns were self-reported by the participants in an interview with clinician experts. The final group assignment was made by multidisciplinary consensus (neuropsychologists, psychiatrists and neurologist) after neuropsychological evaluation. Possible confounders of SCD such as medication, psycho-affective disorders or other relevant medical conditions were dismissed by the clinicians. Following the recommendations made by the SCD-I-WG all subjects were older than 60 at onset of SCD and this occurred within the last 5 years.

The exclusion criteria employed in this study were the followings: (1) history of psychiatric or neurological disorders or drug consumption that could affect MEG activity such as cholinesterase inhibitors, (2) evidence of infection, infarction or focal lesions in a T2-weighted scan within 2 months before MEG acquisition (3) a modified Hachinski score equal to 5 or higher, (4) a GDS-SF score equal to 5 or higher, (5) alcoholism, chronic use of anxiolytics, neuroleptics, narcotics, anticonvulsants or sedative hypnotics. All participants were between 65 and 80 years old. Besides, we conducted additional analysis to rule out other possible causes of cognitive decline such as B12 vitamin deficit, diabetes mellitus, thyroid problems, syphilis, or Human Immunodeficiency Virus (HIV).

All participants signed an informed consent prior to study enrollment. This study was approved by the Clínico San Carlos Hospital ethics committee and the procedure was performed in accordance with approved guidelines and regulations.

### MEG recordings

Neurophysiological data was acquired by using a 306 channel (102 magnetometers, 204 planar gradiometers) Vectorview MEG system (Elekta AB, Stockholm, Sweden), placed in a magnetically shielded room (VacuumSchmelze GmbH, Hanau, Germany) at the “Laboratory of Cognitive and Computational Neuroscience” (Madrid, Spain). All recordings were obtained in the morning, while subjects were sat comfortably, resting awake with eyes closed. Four minutes of MEG signal was acquired for each subject.

Head shape was obtained by using a three-dimensional Fastrak digitizer (Polhemus, Colchester, Vermont). Three fiducial points were acquired (nasion and left and right preauricular points) and at least 300 points of the surface of the scalp. In addition, four head position indication (HPI) coils were placed on the subjects scalp, two in the mastoids and two in the forehead. HPI coils’ position was also acquired using the Fastrak device, in order to provide continuous head position estimation during the recording. Finally, two vertical electrooculogram electrodes were placed above and beneath the left eye of the participants to capture eye blinks and movements.

MEG data was acquired using a sampling rate of 1000 Hz using an online anti-alias filter of [0.1 330] Hz. Recordings were filtered offline using a tempo-spatial filtering algorithm (tSSS, correlation window 0.9, time window 10 seconds)^37^ to eliminate magnetic noise originated outside the head and compensate for head movements.

### MRI acquisition

A T1-weighted MRI was available for each subject, acquired in a General Electric 1.5 Tesla magnetic resonance scanner, using a high-resolution antenna and a homogenization PURE filter (Fast Spoiled Gradient Echo sequence, TR/TE/TI = 11.2/4.2/450 ms; flip angle 12°; 1 mm slice thickness, 256 × 256 matrix and FOV 25 cm). Hippocampal volumes were measured as anatomical evidences of brain atrophy characteristic for MCI and AD^38^. MRI images were processed with Freesurfer software (version 5.1.0) and its specialized tool for automated cortical and subcortical segmentation^39^ in order to obtain the volume of several brain areas. Finally, hippocampal volume was normalized with respect to the overall intracranial volume (ICV) to account for differences in head volume over subjects.

### MEG Signal preprocessing

Ocular, muscular and jump artifacts were first identified using an automatic procedure from the Fieldtrip package^40^, and then visually confirmed by a MEG expert. The remaining data was segmented in 4 seconds epochs of artifact-free activity. Only magnetometers data were used in the subsequent analysis. Subjects with at least 15 clean epochs were selected for further analysis (47.6 ± 7.3 epochs in the no SCD group, 46.2 ± 9.4 epochs in the SCD group, 42.2 ± 7.0 epochs in the MCI group, mean ± standard deviation). There was no significant group effect regarding the number of trials (p = 0.64). In addition, an ICA-based procedure was employed to remove the electrocardiographic component.

### Source reconstruction

Artifact-free epochs were pass-band filtered between 2 and 45 Hz, in order to remove both low frequency noise and network line artifact. The epochs were padded with 2 seconds of real signal from both sides (2000 samples) prior to the filtering to prevent edge effects inside the data.

The source model consisted of 2459 sources placed in a homogeneous grid of 1 cm in MNI template, then linearly transformed to subject space by warping the subject MRI into the MNI template. The leadfield was calculated using three-shell Boundary Element Method (using brain-skull, skull-scalp and scalp-air interfaces) generated from the T1 MRI using Fieldtrip package and OpenMEEG software^41^. Lastly, a Linearly Constrained Minimum Variance (LCMV) beamformer^42^ was employed to obtain the source time series by using the computed leadfield and building the beamforming filter with the epoch-averaged covariance matrix.

### Spectral analysis

The estimated spatial filters were employed to reconstruct the source-space time series for each epoch and source location. MEG power spectra were calculated for every clean epoch with Fieldtrip toolbox. A multitaper method with discrete prolate spheroidal sequences as tapers and 0.5 Hz smoothing for frequencies between 2 and 45 Hz with 0.5 Hz steps was employed. Then the obtained power was normalized with the overall power in [2–45] Hz, thus obtaining the values of the relative power spectra for each frequency step, source and subject.

In order to calculate alpha band power in the most representable and comprehensive way for our sample we manually identified the individual alpha frequency (IAF) for every participant as the most prominent alpha peak in the average power spectrum over occipital and temporal channels. The average IAF in the study sample was 9.4 Hz. Then according to Klimesch considerations about alpha band width^43^ we set it from 6.9 Hz to 11.4 Hz (i.e. IAF − 2.5 Hz–IAF + 2 Hz). Alpha relative power for every source and subject was then computed as the sum of the relative power within the alpha range. Besides, we inspected any other possible power group level effect in theta [4 6.9 Hz] and beta [12 30 Hz] bands following the same procedure and no significant differences were found between groups.

Additionally, alpha peaks parameters were extracted by fitting the source power spectra *P(f*) to a Gaussian peak with power law background, following^24^,^44^.


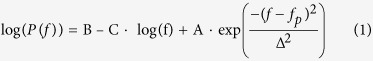


where A, B, C, Δ and f_p_ are fitted with a non-linear least square procedure. A wide fitting range of 4–14 Hz was set. Alpha peak frequencies f_p_ were selected for further analyses.

### Statistical analysis

Statistical analyses in this work was performed in a multi-level approach. Differences across groups regarding neuropsychological scores, alpha relative power and alpha peak frequency were all assessed using an ANCOVA procedure with age as a covariate.

Tukey’s HSD (Honestly Significant Difference) multiple comparisons correction was performed in those neuropsychological tests showing a significant between-groups effect in order to identify which groups’ scores differed.

For source-space alpha peak power and frequency analyses Cluster Based Permutation Tests (CBPT)^45^ were employed for multiple comparisons corrections. The CBPT were executed with 2000 repetitions and a Montecarlo procedure, using always the age as covariate. Alpha level was set to 0.05.

Finally, to assess the structural and behavioural counterparts of the electrophysiological changes we conducted several linear regression models using power and peak frequency values for each subject as predictor variables to estimate neuropsychological performance and hippocampal volume. For these analyses alpha power and peak frequency were averaged across cortical sources. Both variables were included in a single step in the regression as there exists no theoretical definitive justification for ordering the entry of these predictor variables in the model. The equation describing the multiple linear regressions employed is:


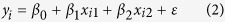


where y is the criterion variable (i.e. neuropsychological performance in each test or hippocampal volume), x_1_ and x_2_ are the variables employed to predict y (alpha relative power and frequency peak respectively), *β*_0_ is the intercept term, *β*_1_ and *β*_2_ are the estimated coefficient for each predictor in the model and *ε* is the error in the prediction of *y*. Then a false discovery rate (FDR^46^, with q = 0.1 was employed to correct for multiple comparisons.

## Results

### Neuropsychological assessment

Neuropsychological performance along with group level differences is listed in Table 1. Of note, the performance of SCD subjects in every neuropsychological measurement laid within a 1SD distance from the average performance of no SCD group. This is crucial to verify their healthy state and rule out any possible objectifiable cognitive decline. Although SCD group scores in Immediate and Delayed Recall were significantly lower compared with no SCD group, each SCD participant performed in the normal range for those subtests. Secondly, MCI patients performed significantly worse than both no SCD and SCD groups in the vast majority of the tests employed which covered: working memory, language, executive functions and praxis.

### Differences in hippocampal volume

Hippocampal volumes obtained with Freesurfer software were compared with a one-way ANCOVA (Table 1). Diagnosis served as the main factor while the age was included in the analysis as a covariate. The effect of diagnosis was significant in the specified contrast (p < 0.01). Pair-wise comparisons revealed a reduction in hippocampal volume for MCI patients against both SCD (p < 0.01) and no SCD groups (p < 0.01). There were no differences in hippocampal volume between SCD and no SCD groups.

#### Differences in alpha peak frequency

Table 1 displays the average IAF per group. As above-mentioned we visually identified IAF values from the posterior sensors for each subject. ANCOVA revealed a significant effect of diagnosis (p < 0.01). Pair-wise comparisons revealed no significant differences between no SCD and SCD groups, thus evidencing a normal alpha peak frequency in SCD participants. MCI subjects presented significantly lower IAF than both SCD and no SCD participants. To further analyze these results and determine the topology of the peak frequency change we performed a source–level comparison of IAF.

#### No SCD vs SCD

Source space comparison of the alpha peak frequency confirmed the previously reported results for the sensor space. No significant differences were found between these two groups.

#### No SCD vs MCI

**Figure 1:**
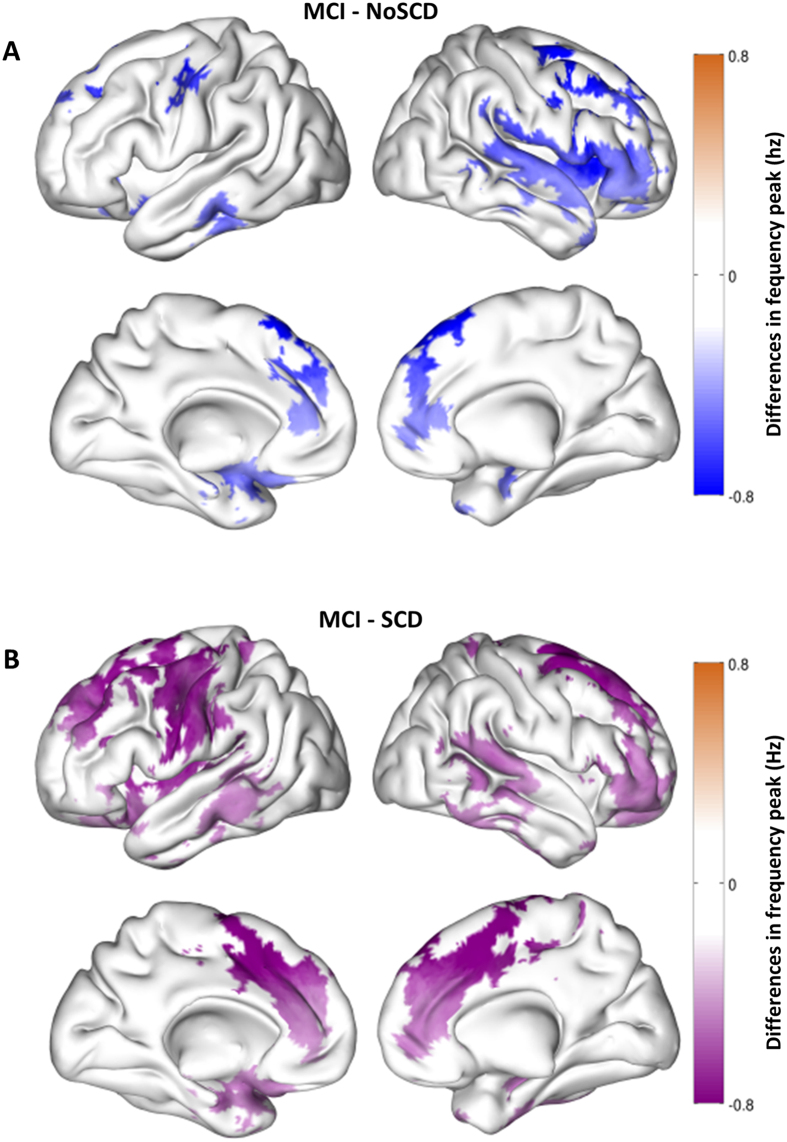
This figure displays the significant differences in the frequency of the alpha peak between groups in the source space. (**A)** (top): Differences between MCI and no SCD. Blue areas represent higher frequency of the alpha peak power in the no SCD group. (**B)** (bottom): Differences between MCI and SCD. Purple areas represent higher frequency of the alpha peak in the SCD group.

There were significant differences between MCI patients and no SCD group regarding alpha peak frequency (p < 0.05). Peak frequency was significantly lower for MCI patients over several areas encompassing bilateral temporal structures such as hippocampus and parahippocampal cortices and both temporal poles, parietal areas including bilateral precentral, postcentral and supramarginal gyri and also bilateral frontal areas (Fig. 1A).

### SCD vs MCI

Statistical testing revealed a slowing in MCI alpha rhythm when compared with the SCD group (p < 0.05). Interestingly, MCI slowing for this contrast exhibited a very similar distribution to the one found for the MCI vs no SCD comparison. Areas with larger differences included bilateral medial temporal structures and bilateral parietal and frontal areas (Fig. 1B).

### Differences in alpha relative power

#### No SCD vs SCD

**Figure 2:**
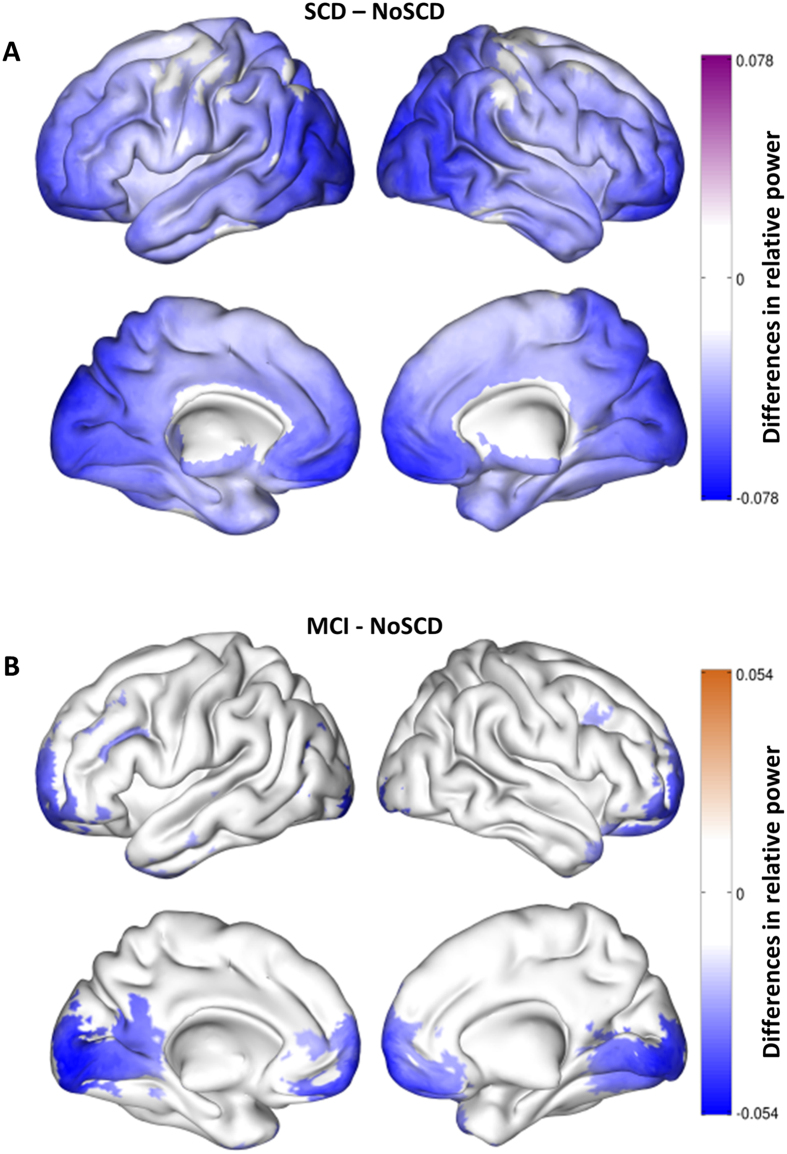
This figure displays the significant differences in alpha relative power between groups in the source space. **(A)** (top): Differences between SCD and no SCD. Blue areas represent higher alpha relative power in the no SCD group. **(B)** (bottom): Differences between MCI and no SCD. Blue areas represent higher alpha relative power in the no SCD group.

Significant differences were found in alpha relative power between no SCD and SCD subjects (p < 0.01). SCD elders exhibited decreased alpha relative power over wide brain regions. The strongest differences were located over bilateral prefrontal areas, bilateral middle and superior temporal lobe, and also bilaterally over calcarine fissure and cuneus in the occipital lobe (Fig. 2A).

#### No SCD vs MCI

Alpha band relative power was significantly diminished in MCI patients when compared with healthy elders without SCD (p < 0.05). These differences were more evident over bilateral occipital areas (Inferior occipital, calcarine, lingual gyrus and cuneus), bilateral prefrontal areas (orbitofrontal cortex, middle and superior frontal gyri) and small portions of the bilateral temporal poles (Fig. 2B).

#### SCD vs MCI

There were no significant differences between SCD participants and MCI group in alpha power after multiple comparisons correction. Thus both groups exhibited a decrease in alpha power with respect to no SCD group with no differences between them in that regard.

### Multiple linear regressions

We performed several multiple linear regressions, using as regressed variable each of the 14 neuropsychological tests and hippocampal volume. The complete set of results is listed in Table 2. After FDR alpha relative power accounted for a significant proportion of the variance in the execution of the following tests: Inverse Digits, Rule Shift Cards, TMTB (both for hits and time) and BNT. The estimated coefficient predicted a better performance in the test with an increase in alpha relative power for all of them. There was a trend toward significance in the same direction for TMTA (time) (p = 0.06). Regarding alpha peak frequency, it explained a significant amount of variance in 11 different models: MMSE, Immediate and Delayed Recall, FAS Semantic, TMTA (both for hits and time), TMTB (hits), Ideomotor Praxis and BNT and hippocampal volume and a trend toward significance for TMTB (time) (p = 0.08). All of the coefficients pointed to a consistent improvement in the execution (i.e. better performance and lower time in TMT) or a higher hippocampal volume with an increase in the frequency of the alpha peak.

## Discussion

The present study seeks to determine whether oscillatory alpha activity measured with MEG sheds light on the discussed consideration of the SCD stage as a part of the AD-continuum. Our results certainly show alterations in the alpha band of SCD participants consistent with AD. Both SCD and MCI groups show common features in their electrophysiological profile. One of the main findings of this study is the reduction in alpha relative power in healthy elders presenting subjective cognitive decline across broad brain regions when compared with age and sex matched counterparts without any cognitive concern. This reduction in alpha relative power was also present in the MCI group. Furthermore, SCD patients did not show any sign of alpha peak frequency slowing, while MCI patients did. In addition, these reductions in alpha power and especially in peak frequency were significantly related with a worse performance in many neuropsychological tests, which confirms their pathological nature. These findings support the interpretation of SCD as the first recognizable stage to date in the AD spectrum and a key phase in the early detection of the Alzheimer’s Disease.

A decrease in MEG power is closely connected with a reduction in the number of active synchronous synapses in a specific frequency range^47^, as MEG signals derive from the spatio-temporal summation of postsynaptic potentials. A plausible hypothesis for this alpha power reduction in SCD participants is that a certain degree of synaptic damage is already present. This would be consistent with the fact that Aβ levels may increase in the brain decades before any plaque deposition or cognitive symptom appears^48^. Furthermore, Aβ has been shown to induce neuronal death through the activation of astrocytes with a potential mechanism involving nitric oxide^49^ and also through neuro-inflammatory responses^50^. In fact, amyloid-β deposition correlates with cortical atrophy over broad brain areas including hippocampus, medial frontal, parietal areas, and lateral temporoparietal cortex in elderly population presenting SCD^51^. These findings support the widespread distribution of the alpha power decrement found in SCD in our work. In addition, elders with SCD accumulated more Aβ over frontal areas as reported in recent PET-PIB studies^52^. In the present study SCD participants exhibited largest power reduction over frontal areas, which is in agreement with the above mentioned studies. FDG-PET studies also reinforce this early synaptic damage hypothesis as elders with SCD were found to exhibit hypometabolism in AD typical areas (for a review see, ref. 53). Regarding electrophysiological studies in SCD, to the best of our knowledge the only study to date reporting spectral power resting state changes in SCD reported an overall increase in absolute alpha power, especially over frontal sensors^28^. However, their results cannot be directly compared with the present work given that authors did not normalize alpha power and especially due to discrepancies in sample recruitment. While in our study SCD subjects showed no cognitive impairment, in the above commented study SCD group performance was apparently affected in every cognitive domain they examined, which makes their sample barely comparable with ours and less representative of the SCD stage.

MCI patients also showed a decrease in alpha relative power compared with healthy elders without subjective cognitive decline in our sample. Reduction in alpha band power in MCI has been largely reported in the literature as one of the most robust and consistent changes in the MCI power spectrum^21^,^26^. However the present work adds valuable spatial information as MEG data was reconstructed in the source space.

SCD and MCI patients showed a comparable decrease in alpha relative power as no significant differences emerged from this comparison. However, SCD’s power reduction seemed to affect broader brain regions than the decrease exhibited by MCI group. A possible explanation for this finding might be related to the 1/f shape of the human power spectrum (i.e. power increases as the frequency decreases). This fact along with the alpha frequency slowing in MCI patients could attenuate the differences in alpha power between MCI and no SCD participants, as MCI alpha peak is localized in a slightly lower frequency range. The fact that the areas showing largest alpha decrease in SCD participants (i.e. frontal and occipital areas) were similar to those where MCI exhibited alpha relative power reduction seems to support this explanation.

SCD group did not show any slowing of their alpha peak frequency. Furthermore, both SCD and no SCD elders showed a significant higher alpha peak frequency compared to MCI patients. Previous studies have reported this slowing pattern for MCI^24^ and AD both compared to healthy controls^54^. However, as far as we know this is the first study addressing the exact frequency of the peak for multiple brain regions in SCD elders. Furthermore, brain areas affected by this slowing in MCI patients were almost exactly the same in the two comparisons, against both SCD and no SCD. It is worth noting that the regions showing substantial slowing highly overlapped with Default Mode Network (DMN) areas: these included medial prefrontal areas, lateral temporal cortex, hippocampus formation and parietal areas. Disruption of the DMN has been previously reported with MEG, and it has been proven to relate to structural damage in white matter tracts in MCI patients^55^. While it is important to point out that our analysis does not capture long range connectivity, our results show a significant decrease of the alpha peak frequency in areas closely linked to the DMN, evidencing in these same regions some degree of synaptic disruption. Cortico-thalamo-cortical models have been employed to simulate changes in the alpha band^56^ reported that a decrease in excitatory firing rate and an increase in inhibitory firing rate produced a slowing of the alpha peak. Alpha peak slowing is also related to a reduction in the number of active synapses in the thalamic nuclei^57^. This could suggest a significant synaptic damage in the MCI stage, for which an alpha slowing has been found.

Although exhibiting a pronounced reduction in alpha relative power, elders with subjective cognitive decline did not show evidences of slowing in their alpha peak, thus being comparable to healthy elders without cognitive concerns in this regard. These results altogether seem to draw a continuum in the AD with respect to alpha band changes. Accordingly, the first sign of the AD pathology in the spectral profile would be a reduction in alpha relative power, which might be related, as mentioned earlier to some degree of synaptic dysfunction. It would be later in the AD-continuum, in the MCI stage, where the slowing of the power spectrum would take place. Several studies have revealed that axonal damage markers are already present in the MCI stage^55^,^58^. According to Buzsaky^47^ this impairment in axon conductance among other parameters, limits the capability of neurons to oscillate in higher frequency rhythms, which could in part explain the slowing we found in the MCI group. Furthermore, this progressive pattern of disruption in the alpha band conforms to thalamo-cortico-thalamic network simulations. Variation of synaptic connections in the thalamic module shows limited effect on the dominant frequency of the alpha band but systematically affect alpha power^57^. In addition, these simulations showed that changes in alpha frequency are much smaller than changes in alpha amplitude^56^, which could make them undetectable in the very early stages of AD.

We studied how alpha band disruption is associated with cognitive changes in our sample. As we expected, both the decrease in alpha relative power, and the slowing of the peak predicted a worse performance in a variety of cognitive domains such as working memory, executive functions, praxis and language. Interestingly, the frequency of the peak demonstrated higher predictive power in multiple tests. This might be due to the fact that changes in alpha power appear in the SCD stage, where some kind of compensation could be still present, as no neuropsychological deficits can be observed. Therefore even though alpha power showed a significant association with performance, the slowing of the peak seems to be intimately related with cognitive decline, as both symptoms appear in the MCI stage.

Hippocampal volume comparisons only revealed a significant reduction in the MCI patients. There are two alternative interpretations of this result. On the one hand, low sensitivity of the automatic segmentation procedures on 1,5T MRI images to detect hippocampal reduction in SCD has been recently highlighted^59^. This study reported hippocampal reduction in SCD with a 3 T system and manual segmentation. On the other hand, there are also studies reporting no hippocampal reduction in SCD^14^. An alternative interpretation is that MEG is able to detect functional alterations in the brain network before structural damage takes place. A 93.3% sensitivity predicting MCI converters has been achieved using spectral analysis of MEG data^60^, underscoring MEG capability of detecting early signs of AD pathology in the brain.

An ever-increasing number of studies point out that SCD is the first identifiable stage of the AD-continuum. In this line, the alpha power reduction could constitute a marker of early AD pathology in the SCD stage which would be followed by a decrease in the frequency of alpha peak in latter stages. These findings have an important clinical meaning since they point out an early synaptic dysfunction in the absence of a sensitive neuropsychological parameter. Thus, a non-invasive technique as MEG could be a good biomarker to detect early neuropathology in the AD-continuum. However, further research is required to confirm this pattern and more importantly to specify and concretize the optimal definition of SCD to reduce inconsistency in the literature.

## Additional Information

**How to cite this article**: López-Sanz, D. *et al.* Alpha band disruption in the AD-continuum starts in the Subjective Cognitive Decline stage: a MEG study. *Sci. Rep.* **6**, 37685; doi: 10.1038/srep37685 (2016).

**Publisher's note:** Springer Nature remains neutral with regard to jurisdictional claims in published maps and institutional affiliations.

## Figures and Tables

**Table 1:** Demographic, neuropsychological and neurophysiological data for each group: No-SCD (N = 39), SCD (N = 41) and MCI (N = 51).

	Mean ± SD			p-values		
	NoSCD	SCD	MCI	NoSCD vs SCD	NoSCD vs MCI	SCD vs MCI
Age	70.4 ± 3.7	71.6 ± 4.5	73 ± 3.7	NS	0.005	NS
Gender (M-F)	12–27	9–32	22–29	—	—	—
Education (years)	14.5 ± 5.5	14.2 ± 5.8	9.5 ± 4.9	NS	1.6 × 10^−4^	3.2 × 10^−4^
GDS	0.9 ± 1.1	1.4 ± 1.2	2.7 ± 2.1	NS	2.4 × 10^−6^	3.3 × 10^−6^
FAQ	0 ± 0	0.1 ± 0.5	1.4 ± 1.7	NS	1.8 × 10^−6^	6.3 × 10^−6^
MMSE	29 ± 1.1	28.9 ± 1.1	27.4 ± 2	NS	4.8 × 10^−5^	2.4 × 10^−6^
Direct Digit	8.5 ± 1.9	8.8 ± 2.1	7.1 ± 2.1	NS	0.011	6.5 × 10^−5^
Inverse Digit	6.2 ± 2.1	5.7 ± 2	4.4 ± 1.5	NS	7.5 × 10^−5^	0.006
Inmediate recall	42.7 ± 11	36.2 ± 11.6	17.4 ± 9	0.032	9.6 × 10^−10^	9.6 × 10^−10^
Delayed recall	26.3 ± 8	21.2 ± 8.7	6.5 ± 7.8	0.032	9.6 × 10^−10^	9.6 × 10^−10^
Rule Shift Cards	3.5 ± 0.7	3.2 ± 1	2.5 ± 1.3	NS	0.002	0.038
FAS-phonemic	14.4 ± 4.7	12.2 ± 3.8	9.1 ± 4.1	NS	1.6 × 10^−7^	0.003
FAS-semantic	18.4 ± 3.4	17 ± 2.7	13.1 ± 3.5	NS	1 × 10^−9^	2.9 × 10^−7^
TMTA (hits)	24 ± 0.2	23.9 ± 0.5	23.9 ± 1.1	—	—	—
TMTA (time)	48.2 ± 19.6	54.4 ± 22.1	77.1 ± 32.4	NS	2.5 × 10^−5^	3.8 × 10^−4^
TMTB (hits)	23.2 ± 3	22.1 ± 3.4	19.7 ± 6.1	NS	0.006	0.052
TMTB (time)	97 ± 43.5	126.5 ± 67.2	211.7 ± 101.5	NS	1.9 × 10^−8^	8.1 × 10^−6^
Idemotor praxis	7.8 ± 0.6	7.5 ± 0.9	7.1 ± 1.2	NS	0.045	NS
BNT	53 ± 8.7	50.9 ± 6.3	44.7 ± 8.6	NS	5.9 × 10^−5^	0.002
Hippocampal vol	5 × 10^−3^ ± 5 × 10^−4^	5 × 10^−3^ ± 4 × 10^−3^	4 × 10^−3^ ± 7 × 10^−4^	NS	3.5 × 10^−4^	2.2 × 10^−4^
IAF	9.8 ± 0.9	9.6 ± 0.9	9 ± 0.9	NS	2.7 × 10^−4^	0.010

The left part of the table shows mean ± standard deviation of the demographic and clinical measurements. The right part shows ANOVA p-values after multiple comparisons when the p-value for the factor is lower than 0.05, otherwise a hyphen is shown; NS, p-value for the factor was greater than 0.05. GDS, Geriatric Depression Scale-Short Form; FAQ, Functional Activity Questionnaire; MMSE, Mini Mental State Examination; TMTA, Trail-Making Test part A; TMTB, Trail-Making Test part B; BNT, Boston Naming Test; IAF, Individual Alpha Frequency averaged over posterior sensors.

**Table 2:** Multiple linear regression models for a set of neuropsychological and neurophysiological measurements.

Criterion	Estimate ± SE		p-values	
	Alpha power	Peak frequency	Alpha power	Peak frequency
MMSE	−0.71	0.47	NS	0.015
Direct Digit	1.63	0.18	NS	NS
Inverse Digit	7.28	0.34	0.032	NS
Inmediate recall	34.67	4.29	NS	0.016
Delayed recall	25.99	3.81	NS	0.006
Rule Shift Cards	4.10	0.42	0.044	0.006
FAS-phonemic	7.99	0.32	NS	NS
FAS-semantic	11.83	1.09	NS	0.019
TMTA (hits)	0.44	0.22	NS	0.016
TMTA (time)	−90.72	−8.72	NS	0.010
TMTB (hits)	18.56	2.02	0.019	2.9 × 10^−4^
TMTB (time)	−326.4	−19.4	0.043	NS
Idemotor praxis	2.35	0.30	NS	0.009
BNT	28.86	2.41	0.052	0.019
Hippocampal vol	−1.1 × 10^−3^	2.2 × 10^−4^	NS	0.008

Each row shows the results for a regression model using the criterion as the predicted variable and alpha power and the frequency of the peak as the predictor variables. Coefficients for each predictor are listed. The right part shows p values for each significant coefficient and ‘NS’ stands for not significant after FDR (q = 0.1); MMSE, Mini Mental State Examination; TMTA, Trail-Making Test part A; TMTB, Trail-Making Test part B; BNT, Boston Naming Test.
